# Polymethyl methacrylate cure time in simulated in vivo total knee arthroplasty versus in vitro conditions

**DOI:** 10.1186/s13018-021-02790-y

**Published:** 2021-10-20

**Authors:** Daniel A. Funk, Quang-Viet Nguyen, Michael Swank

**Affiliations:** 1 c/o Cincinnati Ortho Research Institute, 500 E Business Way, Sharonville, OH 45241 USA; 2 RFA Systems, LLC, 24790 Gypsum Way, Aldie, VA 20105 USA

**Keywords:** Cement temperature, Cement cure time, Total knee arthroplasty cement cure, Cementing total knee arthroplasty, Cement temperature sensor

## Abstract

**Background:**

The present means of confirming the cure of intra-operative polymethyl methacrylate (PMMA) cement are to wait for the remainder cement to harden. To our knowledge, there is no available technique to determine the precise moment of cure for in-vivo cement beneath the tibial tray. This study uses a novel means to determine cement curing time in two environments. One environment represents the operating theater, and the other environment attempts to model cement conditions under the tibial tray during surgery.

**Materials and methods:**

We determined the temperature-versus-time plot of cement curing using the following two temperature sensors: one in a simulated implanted tibial tray and another in the remainder cement. We performed 55 tests using dental methyl methacrylate cement mixed in the same ratio as the orthopedic cement. To simulate in vivo conditions, a simulated stainless-steel tibial tray was implanted on a cancellous bone substitute (Sawbones, Vashon Island, WA, USA) using standard cement technique and subsequently placed in a 90°F (32.2 °C) circulating water bath. We positioned a temperature sensor in the cement mantel and positioned a second sensor in a portion of the remaining cement. The temperature from both sensors was measured simultaneously, beginning at 5 min after mixing and continuing for 20 min. The first derivative of the temperature provided the precise curing time for each condition. We analyzed the results of 55 repeated experiments with an independent samples *t*-test.

**Results:**

With the described technique, we were able to accurately determine the moment of cure of the cement beneath the simulated tray. There was a mean difference between cure time of 5 min and 26 s (*p* value < 0.001) between the two conditions.

**Conclusions:**

We validated that our technique was successful in determining the precise time to cure in two different environments.

**Level of evidence:**

This was not a clinical trial and did not involve patients as such the level of evidence was Grade A: Consistent 1 and 2.

## Introduction

**Fig. 1:**
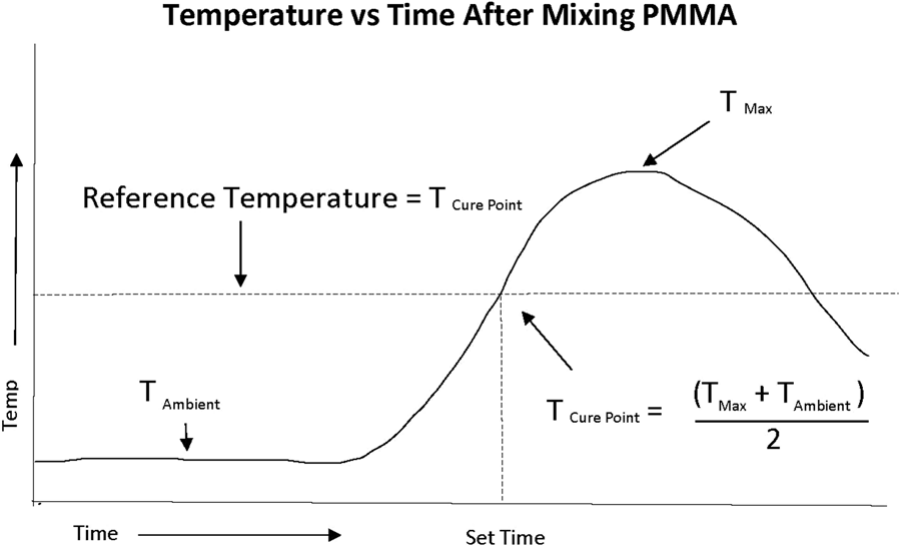
The American Standard for Testing Material (ASTM) PMMA Cure

Acrylic bone cement has been used in orthopedic surgery for over 50 years and is the standard of care for fixation of total joint arthroplasty. After mixing the liquid monomer with the powdered polymer, the cement is converted from liquid to solid by an exothermic reaction. The duration to full polymerization is variable and depends on multiple factors, including temperature and humidity. The American Standard for Testing Material (ASTM) Designation F452-08 [1] specification for full cement curing in a testing environment is based on the temperature of the cement (Fig. 1). The cure temperature (*T*_cure_, i.e., the temperature at which the cement is considered fully cured) is halfway between the maximum temperature of the cement during curing (*T*_max_) and the initial ambient temperature of the cement (*T*_ambient_).

Presently, cement curing during implant surgery is determined either by palpating the cement edge or allowing the remainder cement to harden in vitro. Both methods are imprecise and unscientific. To clinically study intraoperative questions relating to cement cure, such as cement type, viscosity, mixing means, cement temperature, antibiotic inclusion requires the ability to precisely determine the time of cure of the in vivo cement.

Furthermore, the ability to intra-operatively determine cement cure has become more important with recent investigations regarding the causes of aseptic loosening of total knee tibial implants. It has been proposed that lipid infiltration between the tibial tray and the cement interface prevents the cement from interdigitating with the undersurface of the tibial tray, resulting in an area of de-bonded cement. Knee motion prior to full cure of the tibial tray cement can hydraulically wick lipids into the cement–tray interface [2–4]. Consequently, accurate determination of the curing duration of cement under the tibial tray during total knee arthroplasty is important.

This study aims to describe a new technique which can accurately determine cement cure beneath a simulated in vivo tibial plate. To validate that technique a comparison between simulated in vivo and in vitro conditions will be performed. The difference of the time to cure for both conditions will be statistically analyzed. It is proposed that the simulated in vivo cement will cure before the in vitro cement. Confirmation of this finding would indicate that this technique is providing accurate information on the cure point of the cement under the simulated tibial plate.

## Materials and methods

**Fig. 2:**
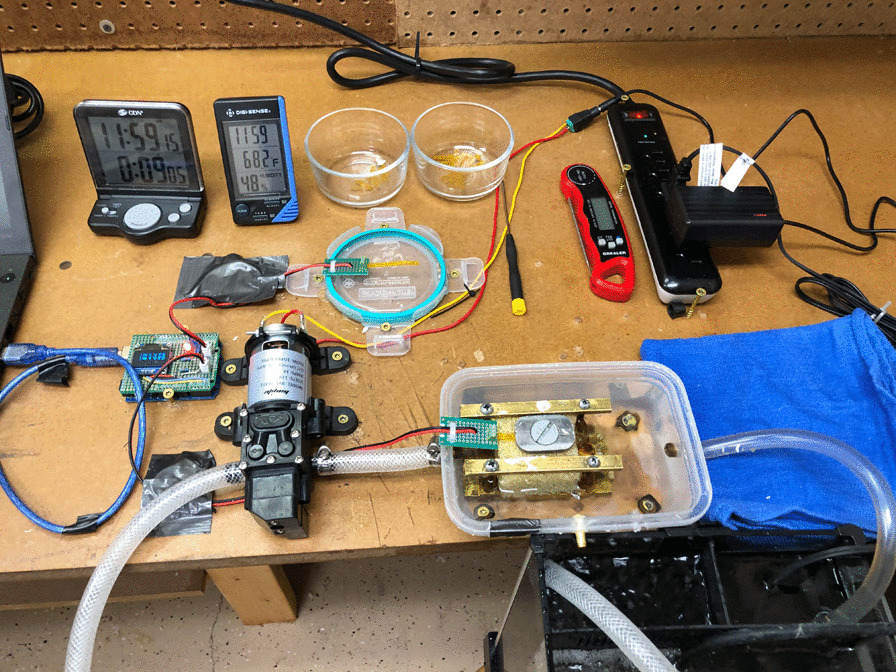
Cement curing and testing station

A 2-inch (5.08-cm) square cube of Cellular Foam, 7.5 PCF, 40 mm (Sawbones, Vashon Island, WA, USA) was used to simulate the cut cancellous bone surface of a tibia prepared for tibial implantation (Fig. 2). A 3/8-inch (0.95-cm) tunnel was drilled through the center of the bone cube from superior to inferior surfaces. A second tunnel that was 0.25 inch (0.64 cm) in diameter was drilled through the bone cube 0.5 inch (1.25 cm) from the bottom of the cube lengthwise and was oriented along the water flow. The foam bone was placed into a brass fixture that secured it at a constant height and then the fixture placed into the water bath.

The tibial implant tray substitute consisted of a 1 × 1.5-inch (2.54 × 3.81-cm) plate composed of 4-mm-thick 316 stainless steel. The bone-facing side of the tray was grit-blasted so that the roughness of the surface finish approximated a standard cemented implant. A 1-inch-long (2.54-cm), 3/8-inch-diameter (0.95-cm) tapered 316 stainless steel stem was attached to the bone-facing side of the tray.

We recognize that the testing implants are significantly smaller and of a different material then standard TKA tibial tray implants. It was not feasible to use real tibial implants given the cost and availability. Our purpose was to prove the usefulness of a new technique for determining cement cure under an orthopedic plate, we believe that the size and material of the test plates would not affect the outcome if the same structure of tray was used for all the experiments.

The water bath consisted of a 3-gal (11.36-L) fish aquarium with a submersible 500-W temperature controller (Hygger HG-921; Hygger, Bantian Group Business Central Longgang District, Shenzhen City Guangdong Province China) calibrated to 0.1 °F accuracy. A submersible 2.9-W filter pump rated at 220 L/h was placed in the tank. The pump outlet was connected to plastic inflow tubing (inside diameter: 3/8 inch [0.95 cm]), with the water exiting into a three-cup (708-cc) plastic storage container (Snapware, http://www.snapware.com). An outflow opening was placed on the opposite side of the container from the inflow. An outlet pump (Bayite 12 V DC; Shenzhen Bayite Technology Co., Ltd, Hong Ji Hua Yuan, Xi Qu #1–705, Long, Cheng Jie Dao, Shenzhen Guang Dong, China) was attached to the outlet tube (inside diameter: 3/8 inch [0.95 cm]), which fed the water back into the water tank. During the experiment, the plastic container acted as a water bath with water at a constant temperature flowing around the simulated cancellous bone 3/4 inches (1.91 cm) below the top surface of the simulated bone surface. We surmised that the cut end of an in vivo tibia would be at a lower temperature than body temperature due to the surgical exposure. To account for the lower temperature in the distal tibia, we decreased the water bath temperature from normal body temperature by 9% to 90°F (32.2 °C).

It was not possible for us to obtain sufficient orthopedic bone cement to utilize in this study due to cost as well as regulations surrounding it’s use. For that reason, we utilized dental denture acrylic as a substitute for orthopedic cement. Dental cement has the same chemical formulation as the orthopedic acrylic cement and behaves in the same manner during its cure cycle. The two-part mixture consisted of a liquid monomer (Jet Liquid; Lang Dental Manufacturing, Wheeling, IL, USA) and polymer powder (Bosworth Duz-All, 166264 W; Bosworth Company, Midland, TX, USA) and was used for each of the 55 experiments. The dental cement was mixed in the same proportion by weight as the orthopedic cement (20-g polymer powder to 10-g monomer liquid). We measured the weights of the components on a digital scale (CGOLDENWALL High Precision Lab Digital Scale Analytical Electronic Balance Scales 0.01-g Calibrated (5000 g, 0.01 g), https://www.amazon.com/Precision-Analytical-Electronic-Laboratory-Calibrated/Model HZ5002) within 0.02 g of the specified weight before mixing. We mixed the cement in an open container with a spatula for two minutes, then allowed the cement to rest for one minute. We monitored the room temperature by a factory-calibrated thermohygrometer (Cole-Palmer Instrument Company, 625 East Bunker Ct., Vernon Hills, IL/ Model # 20,250–30) and maintained the room within one degree of 68°F (20 °C) with room humidity between 45 and 50% during mixing as well as throughout the experiments. Experiments performed outside of these parameters were not included for analysis.

After the rest period, the cement was applied to the top of the Sawbones and undersurface of the tray. A temperature thermistor was placed on the Sawbone side cement and then a small square of thin, porous polyethylene packing material place on the sensor facing the implant to dampen the heat sink effect. The implant was then impacted onto the Sawbones utilizing a Nylon mallet. After impaction, no further pressure was applied to the plate. Temperature controlled water was allowed to circulate around the jig. Subsequently, we inserted a second thermistor into the center of a measured 5-g ball of the remaining cement and placed this cement on the workbench.

We monitored the cement temperature using a thin-film negative temperature coefficient thermistor (NTC: model TT6-10KCB-9-50; TEWA Temperature Thermistors, Ltd, Lublin, Poland) attached to an Arduino programable controller (board model UNO R3; Arduino, Cocos Island). The reproducibility of the system was determined to be − 0.3 °C. The controller had two channels (A and B) that could monitor two TEWA sensors simultaneously. The temperatures of both simulated in vivo and in vitro cement were recorded at a rate of 1/s on an excel spreadsheet.

Our purpose was to compare the time to cure in the two conditions, simulated in vivo and in vitro. To obtain results that were statistically significant, we conducted an independent samples *t*-test. To determine the sample size for the study, we conducted a power analysis (using G*Power version 3.1.9.5 for Mac OS X). To determine the amount of statistical power needed to find a medium effect (Cohen’s *d* = 0.50) for an independent samples *t*-test at the power of 0.80, Cohen (1998)^5^ recommended an overall sample size of 102. This means that 102 participants (51 participants per group) should provide sufficient power to detect an effect.

The testing consisted of 55 separate experiments. Each experiment was prepared and completed in the same manner. During the experiment, recording of the two sensors was performed simultaneously and began at 5 min after the start of the cement mixing and was continued for 20 min (25 min after initiating mixing). The temperature results were captured into a custom Excel spread sheet for analysis. The raw temperature data were smoothed by using a running average of five temperature readings before and after each data point. Further data analysis was performed as discussed in the results section.

## Results

The dental cement temperature plots closely resembled the plots used by ASTM for determining curing, with a period of minimal change proceeding to an exponential increasing temperature and then a decreasing temperature after reaching the maximum temperature (*T*_max_). In our study, we observed consistency in the shape of the temperature plots between experiments for both simulated in vivo and in vitro conditions. Although the plots were similar in shape, the *T*_max_ reached by the two conditions in each experiment varied considerably. The dental cement *T*_max_ for the in vitro condition varied from 66.71°F (34.33 °C) to 109.86°F (43.26 °C) (mean: of 93.79°F [34.33 °C]), and the *T*_max_ for the simulated in vivo cement condition varied from 36.91°F (2.73 °C) to 71.74°F (22.63 °C) (mean: of 49.52°F [9.74 °C]). In both conditions, as the temperature approached *T*_max_, there was a flattening of the temperature plot into an arc-type shape. Consequently, the *T*_max_ in the results could not be precisely determined for use in calculating the cure point (*T*_cure_). Uniformly, the time to cure for the simulated in vivo cement was noticeably shorter than the time to cure for the in vitro cement.

**Fig. 3:**
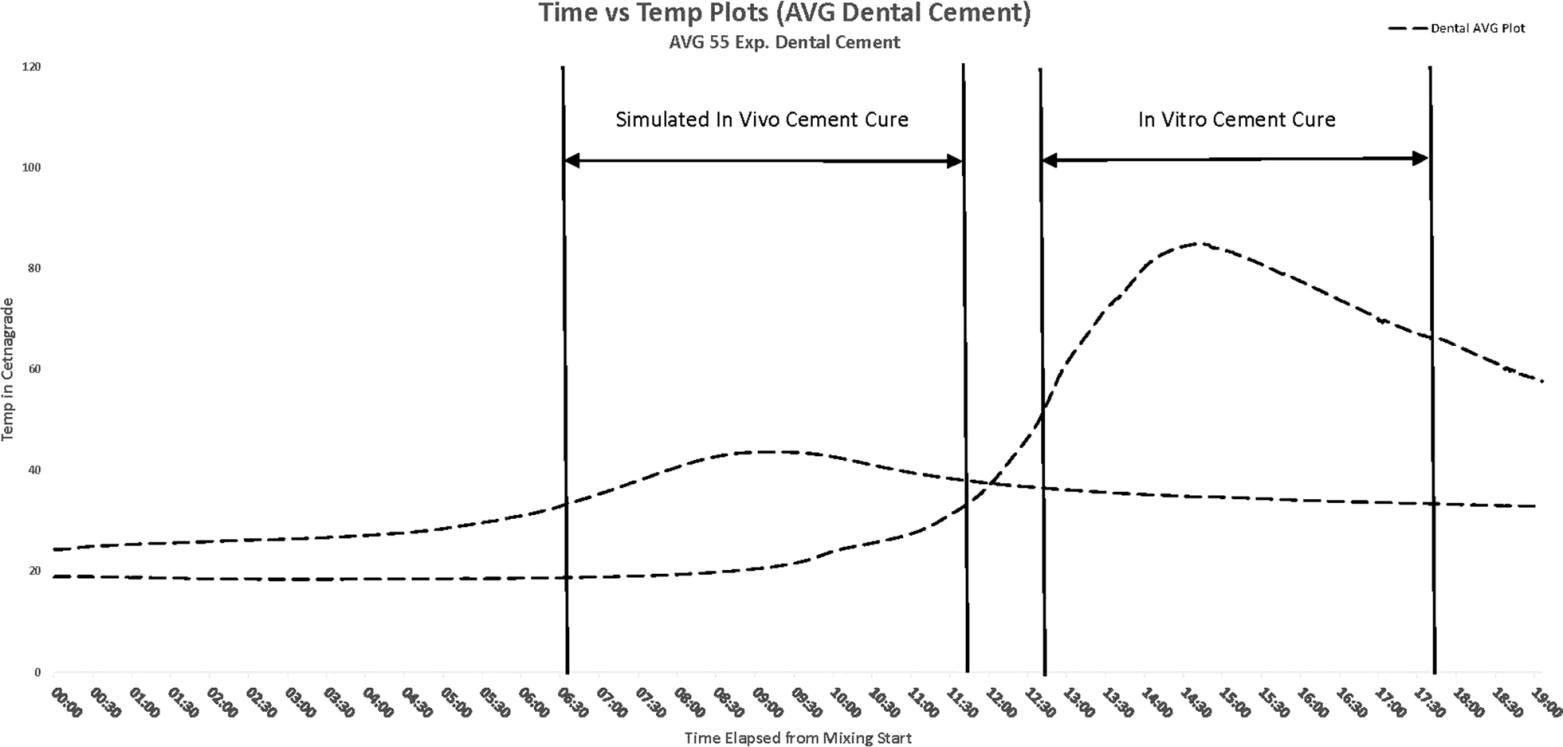
Average time versus temperature plots

The average of all 55 experiments temperature versus time cement plots is shown in Fig. 3. As previously noted, the temperature plots showed significant inconsistency in *T*_max_, implying that it was impossible to use the ASTM definition of cement cure (*T*_cure_). However, based on the shape of the plot, which showed a period of minimal change followed by an exponential increase in temperature and a subsequent reversal of the slope as the cement began to cool, we concluded that using a derivative of the temperature plot would allow us to determine a precise point of cement cure.

**Fig. 4:**
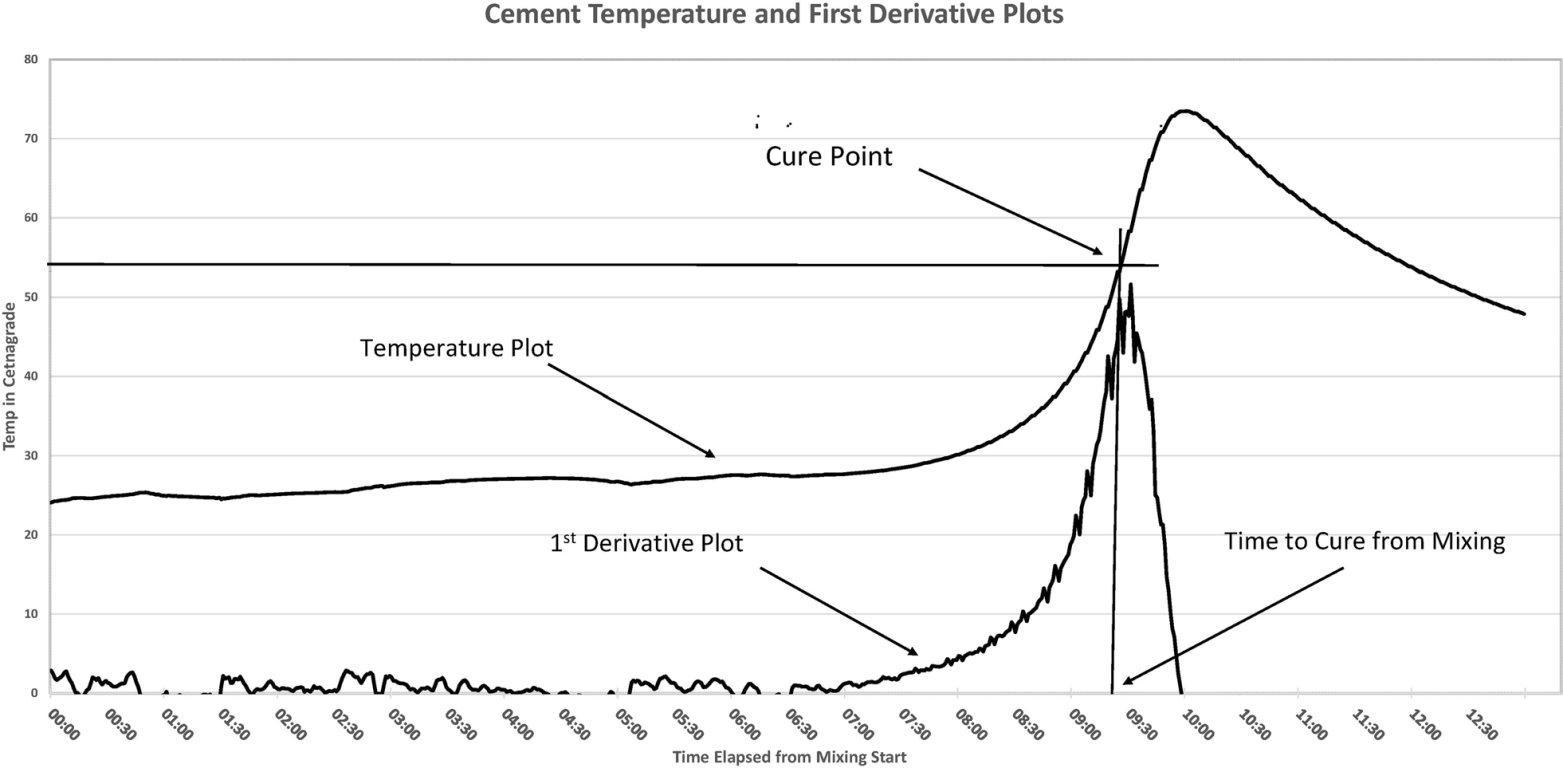
Demonstration of the first derivative

The first derivative is a line tangent to a point on the plot and indicates the instantaneous rate of change in a plot line. As the change in the cement temperature increases, the temperature plot rate of change increases correspondingly. Consequently, the faster the rate of change in the cement temperature, the greater the value of the first derivative. A positive first derivative value means that the temperature is increasing, while a negative first derivative indicates that the cement is cooling. At that inflection point between heating and cooling, the chemical reaction is complete, and the cement can be considered cured. Figure 4 is an illustration demonstrating this principle Notably, in this figure, the time of inflection point is roughly halfway between the ambient temperature and the maximal temperature, corresponding closely with the ASTM guidelines to determine cement cure (*T*_cure_).

Using the first derivative (d*Y*/d*X*) formula:

$$\left( {\left( {\left( {{\text{Temp}}_{{{\text{now}}}} - {\text{Temp}}_{{{\text{Before}}}} } \right) + \left( {{\text{Temp}}_{{{\text{next}}}} - {\text{Temp}}_{{{\text{now}}}} } \right)} \right)/\Delta {\text{Time}}} \right)/2.$$

we calculated the first derivative value for each of the 55 temperature plots. The peak value of the first derivative for all 55 experiments under both simulated in vivo and in vitro conditions was determined (102 analyzed plots). The time difference between the maximum value of the first derivative for the simulated in vivo condition, subtracted from the in vitro condition, is the time difference between curing in each of the 55 experiments.

We conducted an independent samples *t*-test to evaluate significant differences in cement curing time between in vivo and in vitro cement. The results revealed that the curing time for in-vivo cement (*Mean(M)* = 7.66 [7 min(min) 39 s(sec)], *SD* = 1.07 [1 min 4 s]) was significantly quicker compared to the curing time for in vitro cement (*M* = 13.09 [13 min 5 s], *SD* = 1.18 [1 min 11 s], t [108] = − 25.20, *p* < 0.001 [mean difference = − 5.43 5 min 26 s]). We observed that the effect size for this analysis (*d* = 4.82) exceeded Cohen’s [5] convention for a large effect (d = 0.80). Skew and kurtosis met the criteria for acceptable limits for normality (skewness = 0.039; kurtosis = -1.338; Fields [6]).Table 1 summarizes the results from the independent samples *t*-test.

**Table 1:** Summary of independent samples *t*-test

	t	df	p	Mean difference	Std. error of difference	95% CI	
						Lower	Upper
Curing Time	18.70	108	.000	− 5.21	0.28	− 5.76	− 4.65

The results indicate that the simulated in vivo cement cured at a significantly faster rate than the in vitro cement. The magnitude of this effect was large, suggesting that the difference is important. The significance level (alpha) is the probability of making a type 1 error (i.e., rejecting a true null). A *p* value of < 0.001 indicates that the results are highly significant, and there is < 1 in a thousand chance of making a type 1 error (or being incorrect).

## Discussion

Orthopedic surgeons performing total joint arthroplasty are aware of the thermal characteristics of acrylic bone cement [7–10]. Previous studies on this phenomenon primarily aimed to determine whether the heat generated by the curing cement causes biological damage [11, 12] Simulated in vivo and in vitro studies have been performed to quantify the peak temperature of cement during curing [13–15]. Techniques to lower the maximum temperature of curing cement have been proposed to decrease possible thermal tissue damage [16–18].

Aseptic loosening of the tibial base tray after total knee arthroplasty remains a major cause of revision surgery [19]. Recent focus has been on lipid infiltration under the tibial tray. This infiltration prevents the cement from obtaining a secure mechanical bond on the tibial tray surface. New tibial implant designs have been developed to decrease the possibility of lipid infiltration [20]. Motion of the knee prior to complete cement cure can increase the risk of lipid infiltration and lead to subsequent debonding. Even with the new tray designs, the knee motion prior to cement cure remains a possible source of aseptic loosening. There is an ongoing debate whether certain implants are more susceptible to aseptic loosening or whether a two-batch technique with full cure of the tibial tray before proceeding with the remaining surgery should be the standard of care [21, 22] Thus, clinical confirmation of cement cure before motion is important to the longevity of an implant.

To our knowledge, the use of the polymerizing cement temperature to determine the curing time under an orthopedic implant has not been previously studied. Presently, determining the duration of full cure of the cement intraoperatively is, at best, imprecise. Using the remaining cement to establish the completion of curing has not changed since the early days of arthroplasty. Waiting for the extra cement to harden is a time-honored artistic ritual for surgeons and staff.

A technique to determine when the tibial tray cement is cured would allow for more accurate study of cement behavior in different conditions. Variables such as cement temperature, cement viscosity and antibiotic additions could be evaluated by finding the precise time of cement cure in vivo. From a clinical standpoint, knowing the time of cement cure would allow the surgeon to determine when it is safe to move the knee, potentially decreasing the risk of aseptic loosening. We have demonstrated that using the first derivative of the temperature plot of curing cement is valid in ascertaining the point of full cure (*T*_cure_). We have also shown that it is possible to use the temperature plot to determine the cure point of cement in a simulated operative condition.

This study corroborated the clinical observation that there is a time difference between the two curing points for in vivo and in vitro cement. The average curing time of the simulated in vivo cement was 5 min 26 s quicker than that of the in vitro cement. Since there are multiple factors that affect cement cure times during surgery, we are not saying that in practice the difference between cure of cement under the tibial tray and external cement is of this magnitude. However, it is reasonable to assume that intraoperatively, the in vivo cement may cure significantly before external in vitro cement. As a result, waiting for the in vitro cement to cure during a cemented arthroplasty can add unnecessary time to the surgical procedure. The intra-operative use of a device to confirm cement cure could be used to safely shorten operative time.

There are limitations to this study. First, the simulated in vivo conditions may not accurately reflect the true conditions under the tibial tray during total knee arthroplasty. Multiple factors can alter the temperature plot, including the use of a tourniquet and lavage of the surgical site. To verify our findings, further clinical investigation is necessary.

Second, the study used dental cement as a substitute and, thus, may not truly reflect the behavior of orthopedic cement. Since the curing time of cement is changed by multiple factors intra-operatively, it would be logical to conclude that the time to cure will change depending on the type of cement used. However, we believe that the shape of the temperature vs time plots will maintain the same profile.

This study does provide support that using excess in vitro cement for determining the curing of in vivo cement is valid. This study does not provide information on whether palpating the cement mantel during surgery can be used to determine simulated in vivo cement curing. The study supports the surgical observation that the cement under the tibial tray cures quicker than the remaining in vitro cement.

In conclusion, the temperature-versus-time plot of acrylic bone cement was utilized to determine the cure point (*T*_cure_) of bone cement in two conditions: simulated in vivo and in vitro. The study validated that by using the first derivative of the temperature plot, the accurate cure time of bone cement under a simulated in vivo tibial tray can be determined. Developing a clinical device using temperature sensors to determine tibial tray cement cure during surgery would allow for further investigations into cement behavior. Such a device could also be used to potentially shorten the surgical time of cemented total knee arthroplasty while decreasing the risk of lipid infiltration.

## Data Availability

The datasets used and/or analyzed during the current study are available from the corresponding author on reasonable request.

## Abbreviations

PMMA: Polymethyl Methacrylate
ASTM: American Standard for Testing Material
NTC: Negative Temperature Coefficient

## References

[1] ASTM International. Designation: F451-16, standard specification for acrylic bone cement. West Conshohocken, PA: ASTM International; 2019.

[2] DePuySynthes. Guidance for cementing total knee replacements*.* Warsaw, IN: DePuy Orthopedics; 2018.

[3] Billi F, Kavanaugh A, Schmalzried H, Schmalzried T. Factors influencing the initial strength of the tibial tray–PMMA cement bond [poster number 1854]. ORS annual meeting; 2014.

[4] Maag C, Peckenpaugh E, Metcalfe A, Langhorn J, Heldreth M. Influence of intra-operative lipid/marrow infiltration and intra-operative motion upon cemented tibial implant fixation [poster no. 1788]. ORS annual meeting; 2019.

[5] Cohen J (1988). Statistical power analysis for the behavioral sciences.

[6] Field A (2013). Discovering statistics using IBM SPSS statistics.

[7] Meyer PR, Lautenschlager EP, Moore BK (1973). On the setting properties of acrylic bone cement. J Bone Joint Surg Am.

[8] Dunne NJ, Orr JF (2002). Curing characteristics of acrylic bone cement. J Mater Sci Mater Med.

[9] Li C, Mason J, Yakimicki D (2004). Thermal characterization of PMMA-based bone cement curing. J Mater Sci Mater Med.

[10] Kühn K-D (2014). PMMA cements.

[11] DiPisa JA, Sih GS, Berman AT (1976). The temperature problem at the bone-acrylic cement interface of the total hip replacement. Clin Orthop Relat Res.

[12] Linder L (1977). Reaction of bone to the acute chemical trauma of bone cement. J Bone Joint Surg Am.

[13] Deramond H, Wright NT, Belkoff SM (1999). Temperature elevation caused by bone cement polymerization during vertebroplasty. Bone.

[14] Harving S, Søballe K, Bünger C (1991). A method for bone–cement interface thermometry. An in-vitro comparison between low temperature curing cement Palavit® and Surgical Simplex® P. Acta Orthop Scand.

[15] Vertullo CJ, Zbrojkiewicz D, Vizesi F, Walsh WR (2016). Thermal analysis of the tibial cement interface with modern cementing technique. Open Orthop J.

[16] Li C, Schmid S, Mason J (2003). Effects of pre-cooling and pre-heating procedures on cement polymerization and thermal osteonecrosis in cemented hip replacements. Med Eng Phys.

[17] Koh BT, Tan JH, Ramruttun AK, Wang W (2015). Effect of storage temperature and equilibration time on polymethyl methacrylate (PMMA) bone cement polymerization in joint replacement surgery. J Orthop Surg Res.

[18] Seeger JB, Jaeger S, Bitsch RG, Mohr G, Rohner E, Clarius M (2013). The effect of bone lavage on femoral cement penetration and interface temperature during Oxford unicompartmental knee arthroplasty with cement. J Bone Joint Surg Am.

[19] Lombardi Jr AV, Berend KR, Adams JB. Why knee replacements fail in 2013: patient, surgeon, or implant? Bone Joint J. 2014;96-B:101–104. 10.1302/0301-620X.96B11.34350 25381419

[20] ATTUNE S+™ Technology with Macrolock and Microblast (2016) https://www.jnjmedicaldevices.com/en-US/product/attune-knee-system. Accessed 10 Aug 2020.

[21] Arsoy D, Pagnano MW, Lewallen DG, Hanssen AD, Sierra RJ (2013). Aseptic tibial debonding as a cause of early failure in a modern total knee arthroplasty design. Clin Orthop Relat Res.

[22] Mason J, Behnam Y, Fehring T, Clary C. Simultaneous femoral and tibial cementation negatively effects tibial fixation in total knee arthroplasty [scientific exhibit 15]. AAOS annual meeting, 2018.

